# Spatio-temporal control of asymmetric septum positioning during sporulation in *Bacillus subtilis*

**DOI:** 10.1016/j.jbc.2024.107339

**Published:** 2024-05-04

**Authors:** Katarína Muchová, Jiří Pospíšil, Evelína Kalocsaiová, Zuzana Chromiková, Silvia Žarnovičanová, Hana Šanderová, Libor Krásný, Imrich Barák

**Affiliations:** 1 Department of Microbial Genetics, Institute of Molecular Biology, Slovak Academy of Sciences, Bratislava, Slovakia; 2 Laboratory of Microbial Genetics and Gene Expression, Institute of Microbiology of the Czech Academy of Sciences, Prague, Czech Republic

**Keywords:** *Bacillus*, asymmetric cell division, sporulation, chromosomes, protein-protein interaction, SpoIIE, RefZ

## Abstract

During sporulation, *Bacillus subtilis* forms an asymmetric septum, dividing the cell into two compartments, a mother cell and a forespore. The site of asymmetric septation is linked to the membrane where FtsZ and SpoIIE initiate the formation of the Z-ring and the E-ring, respectively. These rings then serve as a scaffold for the other cell division and peptidoglycan synthesizing proteins needed to build the septum. However, despite decades of research, not enough is known about how the asymmetric septation site is determined. Here, we identified and characterized the interaction between SpoIIE and RefZ. We show that these two proteins transiently colocalize during the early stages of asymmetric septum formation when RefZ localizes primarily from the mother cell side of the septum. We propose that these proteins and their interplay with the spatial organization of the chromosome play a role in controlling asymmetric septum positioning.

Spore formation, or sporulation, in *Bacillus subtilis* is the ultimate response to nutrient deficiency. This developmental pathway is costly in terms of time and energy and thus must be stringently controlled. During sporulation, *B. subtilis* changes its site of division from the midcell to a position close to a cell pole. Asymmetric division then gives rise to two daughter cells of unequal size and different fates. The smaller forespore is subsequently engulfed by the larger mother cell and the two cells cooperate in the formation of a thick proteinaceous shell, the spore coat. In the final stage, the mature spore is released from the lysing mother cell. The spore can then lie dormant indefinitely and germinate when growth conditions improve (1, 2).

The earliest observable differences between sporulating and vegetative cells are changes in nucleoid morphology and in the localization of the Z-ring (comprised of a polymer of the protein FtsZ), which marks the site of the septum (3). Soon after entry into sporulation and before the asymmetric septum forms, the two completely replicated chromosomes form an elongated structure called the axial filament (4, 5). Attachment of the chromosome to the cell poles requires the sporulation-specific protein RacA (6), and these nucleoprotein complexes are tethered to the pole by DivIVA (6, 7, 8). Recently, an additional chromosome anchoring mechanism was discovered, which involves the competence regulator ComN, and the division site selection regulators MinD and MinJ (9).

Once the axial filament is formed, the midcell Z-ring transforms into a helical structure that moves toward each cell pole. This helical structure then splits into two polar spirals and this redeployment of FtsZ culminates in the formation of two separate Z-rings near the two poles (10). This Z-ring redeployment is reinforced by SpoIIE and increased expression of *ftsAZ* from a second sporulation-specific promoter (10). Eventually, one of these two polar Z-rings becomes the site of asymmetric division (11, 12).

The sporulation-specific SpoIIE protein is crucial for proper asymmetric septation and thus the progression of sporulation. SpoIIE is a large membrane protein that consists of three main domains: an N-terminal domain (domain D1, residues 1–330), which is formed by ten membrane-spanning segments; a central regulatory domain (domain D2, residues 331–589), which is thought to be involved in its interaction with FtsZ; and a C-terminal domain (domain D3, residues 590–827), which is a PP2C-type phosphatase (13, 14). SpoIIE has several roles in sporulation. First, it is required for asymmetric septum formation when it is targeted to the polar sites together with FtsZ and colocalizes there with the polar Z-rings to form the so-called E-ring (10, 15). SpoIIE then remains as an integral component of the final asymmetric septum (16, 17). Second, SpoIIE dephosphorylates the antisigma factor antagonist SpoIIAA, thereby activating the first forespore-specific transcription factor, σ^F^ (18, 19, 20, 21). A putative third role is connected with forespore engulfment and peptidoglycan remodeling (22); *ΔspoIIE* cells are defective in sporulation with aberrantly thick asymmetric septa formed at lower frequency (23, 24). Finally, we recently uncovered a fourth role for SpoIIE in the determination of the asymmetric septum position (25).

The Min system is another player in the positioning of the asymmetric septum. It consists of MinCD, the topological factor DivIVA, and the membrane protein MinJ. This system ensures that the Z-ring appears at the cell midpoint during vegetative division (26). To allow asymmetric septum formation, the function of the Min system must somehow be reprogrammed since the Min system proteins DivIVA, MinD, and MinJ do not simply switch off during sporulation but have a role in anchoring the chromosome to the cell poles (6, 7, 8, 9). DivIVA also takes part in the redeployment of FtsZ and SpoIIE from the midcell to the polar positions directly interacts with SpoIIE and is required for the correct compartment-specific activation of σ^F^ in the forespore (27). Although deletion of *minCD* has no obvious effect on sporulation frequency (28), it was recently observed that the position of the septation site is deregulated in the absence of MinCD (25).

RefZ is an additional sporulation-specific protein involved in asymmetric septation. RefZ was initially discovered to promote the repositioning of FtsZ from the midcell to the cell poles at the onset of sporulation (29). In cells lacking RefZ, the shift of FtsZ from midcell is delayed, and this effect is even stronger in cells lacking both RefZ and SpoIIE. RefZ binds to its cognate motifs in DNA (RefZ binding motifs, RBMs), one located near *oriC* and two each on the left and right chromosomal arms (29, 30). The left and right arm, RefZ binding motifs, RBMs lie at the boundary determining the part of the chromosome trapped in the forespore at the time of polar septation (30). Recently, we showed that cells without RefZ position the asymmetric septation site further away from the poles which indicates that RefZ participates in the control of asymmetric septum positioning (25).

Altogether, despite decades of research, the proteins, their interactions, and the mechanisms of action involved in the positioning of the asymmetric septum are still not fully understood. Even though the key role of SpoIIE in this process is unquestionable, it is not clear how SpoIIE fulfills this role. In this work, we focus on interactions of proteins that were shown to influence the sporulation septum site localization, RefZ and DivIVA (25). We find that SpoIIE specifically binds to the negative division regulator RefZ, and we explore this interaction in detail. We also show the temporally and spatially resolved localization of these proteins in the early stages of sporulation. We propose a model in which the interaction of these two proteins is important for the asymmetric septum site determination. We hypothesize that this complex exists only transiently and that once the septum position is determined, RefZ detaches and the septum forms.

## Results

### Bacterial two-hybrid screen identifies RefZ as a new interacting partner of SpoIIE

**Figure 1:**
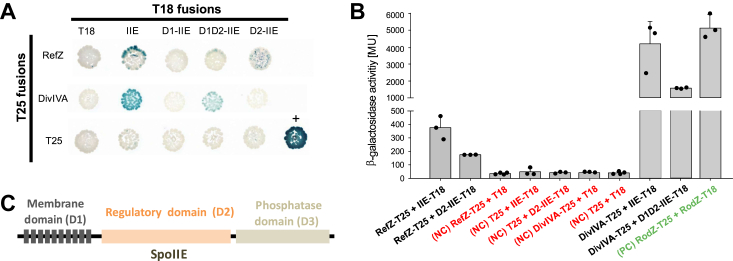
**Interactions of SpoIIE and its individual domains with RefZ and DivIVA.***A*, BACTH reveals a new SpoIIE (IIE) interaction with the sporulation specific regulator RefZ. *Escherichia coli* strain BTH101 (Δ*cya*) was cotransformed with plasmids encoding the indicated fusions to adenylate cyclase fragments T18 and T25. Colonies were spotted on selective plates containing IPTG and X-Gal. The *blue color* indicates a positive interaction between each pair of fusion proteins. The positive control, pKTrodZ+pUTCrodZ, is marked +. *B*, BACTH β-galactosidase activity assays. Normalized β-galactosidase activity expressed in Miller units (MU) is shown. The mean values from each experiment were normalized to the negative-control (NC) values (BTH101 cells coexpressing only T18 and T25 subunits of adenylate cyclase). The values of specific negative controls (only one subunit of adenylate cyclase fused with the respective target; the second subunit remained free) are also shown. Each experiment was performed at least three times independently. The bars represent averages, the dots individual experiments. Error bars represent ±SD. *Red* and *green* text in x-axis description indicates negative (NC) and positive controls (PC), respectively. Note that some of the negative interactions from A) are not included in this assay. *C*, domain organization of the SpoIIE protein: membrane domain (D1), regulatory domain (D2), and phosphatase domain (D3).

Our previous results have shown that the precise localization of the sporulation septum depends on the presence of SpoIIE, RefZ, and Min proteins (25). SpoIIE is a key player in the formation of sporulation septum but also is crucial in the determination of asymmetric septum position. To identify potential direct protein-protein interaction between SpoIIE and DivIVA (Min system protein), and between SpoIIE and RefZ we used a bacterial two-hybrid (BACTH) system (31). We confirmed that SpoIIE interacts strongly with DivIVA in this assay, (Fig. 1, *A* and *B*) and this is in agreement with a previously described interaction between these two proteins at the polar septum (27). Moreover, we found a so far undescribed interaction between SpoIIE and RefZ (Fig. 1, *A* and *B*).

To analyze these interactions in more detail, we prepared individual SpoIIE domain constructs – membrane domain D1 (D1-IIE), membrane domain D1 + the regulatory domain D2 (D1D2-IIE), and the regulatory domain D2 alone (D2-IIE) (Fig. 1*C*). Previously, it was shown that the phosphatase domain D3 (D3-IIE) is self-activated, and therefore it could not be used when combined with the relevant empty vector (32). Results of the BACTH experiments then revealed interactions of the SpoIIE regulatory domain (D2-IIE) with RefZ (Fig. 1, *A* and *B*), and the SpoIIE combined domains (D1D2-IIE) with DivIVA (Fig. 1, *A* and *B*).

All these interactions show that SpoIIE can directly interact with the negative division regulators RefZ and DivIVA as a member of Min system. Consequently, we focused on characterizing the newly identified SpoIIE-RefZ interaction in more detail.

### Cytosolic part of SpoIIE interacts with RefZ in an *in vitro* pull-down assay

**Figure 2:**
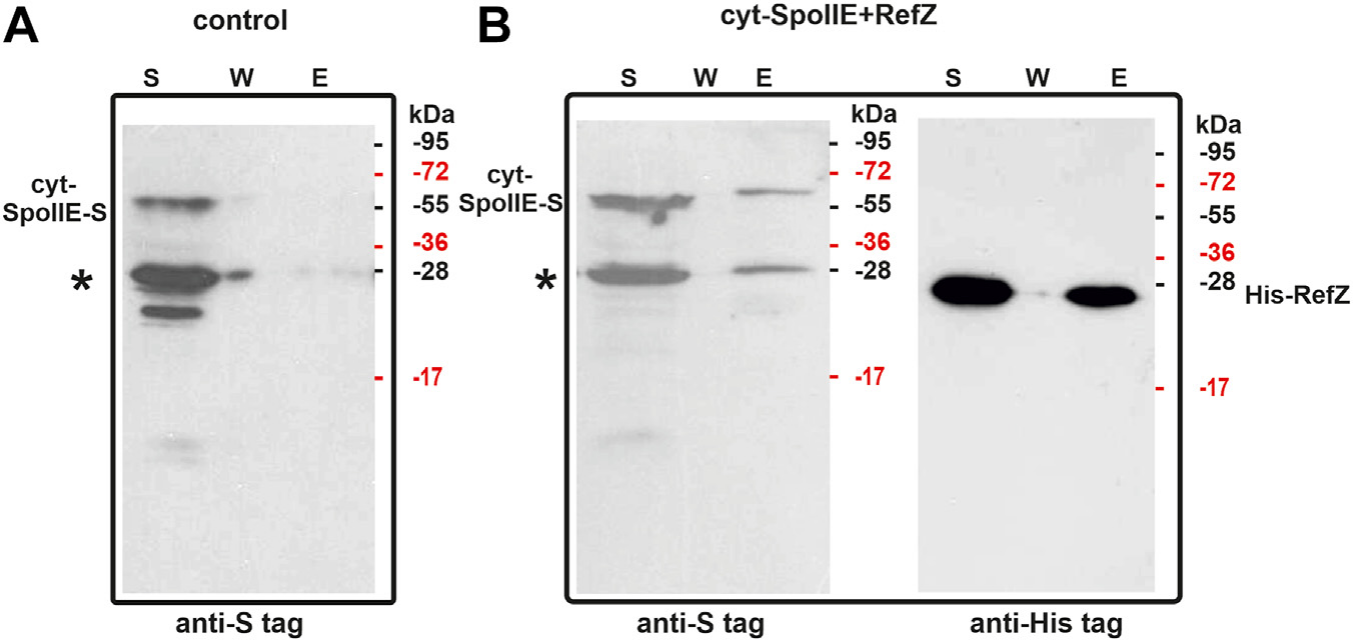
**Interaction of the cytosolic part of SpoIIE (cyt-SpoIIE) with RefZ tested by pull-down assay.***A*, control for unspecific binding of the cyt-SpoIIE-S tagged fusion protein to the Ni^2+^ column. Lysates from the cells expressing only cyt-SpoIIE-S were applied to the column, washed, and eluted with 1 M imidazole (panel *A*, lane *E*). *B*, His-RefZ pulls down cyt-SpoIIE-S from *Escherichia coli* cell lysates on Ni^2+^column (panel *B* lane *E*). Soluble fractions (S) from *E. coli* cells expressing fusion proteins as well as the final wash fraction (W) and eluent (E) were tested by Western blotting using anti-S tag and anti-His tag monoclonal antibodies. A selected part of the protein ladder is shown on the right of the western blots. cyt-SpoIIE-S is partially degraded and the degradation product is marked by *asterisk*. Each lane contains 10 μl of sample prepared as described in Experimental procedures.

The BACTH system does have limitations (33) and additional biochemical methods are therefore needed to verify protein interactions detected using it. We performed *in vitro* pull-down assays, using an *Escherichia coli* strain expressing cyt-SpoIIE-S, the soluble cytosolic part of SpoIIE (D2+D3) with an S-tag fused to its C terminus (34), and *E. coli* strain expressing His-tagged RefZ with His-tag fused to its N terminus (Experimental procedures). We confirmed expression and solubility of these proteins by SDS-PAGE. We then mixed cell extracts to detect the interactions. The mixture of cell extracts cyt-SpoIIE-S+His-RefZ was loaded onto Ni^2+^ column. After several washing steps, the fusion proteins were eluted and subsequently detected by multiplex Western blotting, using anti-His tag and anti-S tag monoclonal antibodies. To test for nonspecific binding of cyt-SpoIIE-S to the column, extract from cells expressing only cyt-SpoIIE-S was loaded onto a Ni^2+^ column as a control: no S-tagged cyt-SpoIIE was detected in elution fractions when produced alone (Fig. 2*A*, lane E). cyt-SpoIIE-S was then pulled down with His-RefZ (Fig. 2*B* lane E) suggesting that the cytosolic part of SpoIIE directly associates with RefZ.

### SpoIIE and RefZ form a complex

**Figure 3:**
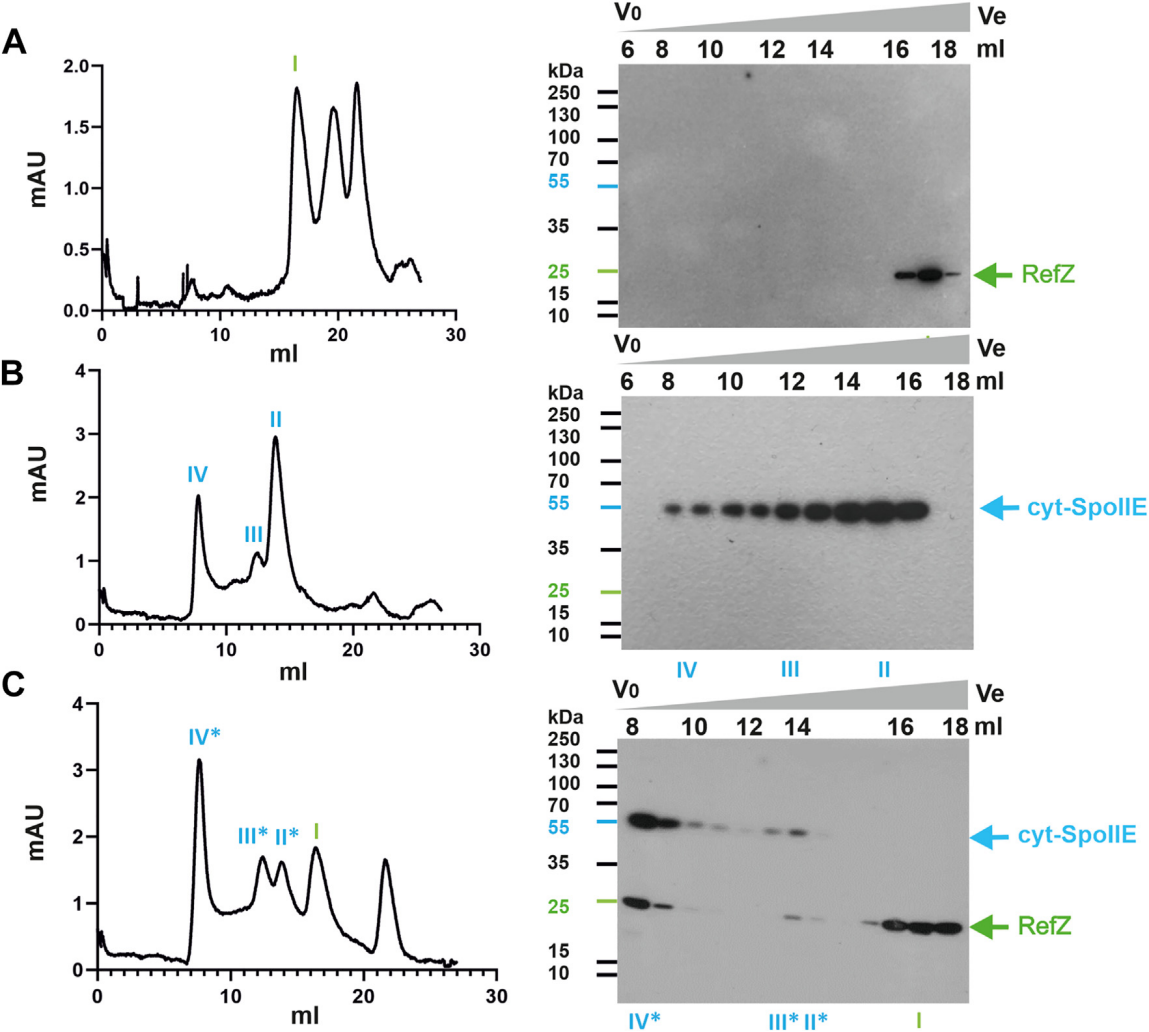
**Interaction of SpoIIE and RefZ as detected by gel filtration assay.** RefZ, cyt-SpoIIE and a mixture of RefZ and cyt-SpoIIE were applied to a Superdex gel filtration column as described in Experimental procedures. Both proteins were His-tagged at the N terminus. Elutions of proteins were performed in the same buffer and 0.5 ml fractions were collected and analyzed by Western blotting using an anti-His monoclonal antibody. *A*, purified RefZ was applied to the column and eluted in a single peak (*I*) corresponding to a RefZ monomer with an apparent molecular weight of 31 kDa as calculated from the calibration curve of this column. The latter two peaks, at higher elution volumes, did not contain RefZ as shown in Western blot in Fig. S1. *B*, purified cyt-SpoIIE was applied to the column and eluted in peaks corresponding to dimers (∼101 kDa, peak II), tetramers (∼205 kDa, peak III), and higher oligomeric species (peak IV). *C*, when RefZ premixed with cyt-SpoIIE was applied to the column, part of RefZ coeluted with cyt-SpoIIE in a peak representing a higher oligomeric species (IV∗). A weak RefZ signal was also detected in the elution fractions corresponding to cyt-SpoIIE tetramers (III∗) and dimers (II∗). This shows that RefZ interacts with cyt-SpoIIE and that a RefZ–cyt-SpoIIE complex is formed. Chromatograms of the eluted proteins are shown on the *left*; mAU represents the recorded absorbance at 280 nm, and elution volume is shown in ml. Western blots using an anti-His antibody are shown on the right-hand side. V_0_ – void volume, V_e_ – elution volume. The lanes from corresponding peaks for cyt-SpoIIE and RefZ (I–IV and II∗–IV∗) from the gel filtration profile are marked in the Western blots. The protein ladder is shown to the left of the Western blots.

To investigate the interaction between SpoIIE and RefZ in more detail, purified cyt-SpoIIE and RefZ were applied to a gel filtration column either separately or mixed. The elution patterns for RefZ, cyt-SpoIIE, and the RefZ+cyt-SpoIIE mixture were monitored by Western blotting. Gel filtration analysis of purified RefZ revealed that RefZ eluted mainly as a single peak with an apparent molecular weight of 31 kDa, corresponding to a RefZ monomer (Fig. 3*A*, Fig. S1). A similar purified RefZ elution profile was observed in a previous study where the majority of this protein was also monomeric (35). However, RefZ binds to DNA as a dimer or tetramer, and these forms are thought to be important for its *in vivo* role. These forms are probably not stable enough to be maintained in the absence of DNA during gel filtration experiments (35).

When cyt-SpoIIE was passed through a gel filtration column alone (Fig. 3*B*), peaks corresponding to higher-order multimers, tetramers (∼205 kDa), and dimers (∼101 kDa) were detected. This is consistent with previous reports where the multimerization of SpoIIE was detected by both gel filtration and analytical ultracentrifugation (21). Tetramers and higher-order mobile oligomeric species of SpoIIE in sporulating *B. subtilis* cells were also recently detected by single-molecule optical microscopy (36). When a mixture of RefZ and cyt-SpoIIE, prepared as described in Experimental procedures, was loaded onto a gel filtration column, part of RefZ coeluted with cyt-SpoIIE in a peak representing higher oligomeric species (Fig. 3*C*). In addition, a weak RefZ signal was also detected in elution fractions corresponding to cyt-SpoIIE tetramers and dimers (Fig. 3*C*). These results show that RefZ interacts with cyt-SpoIIE and that a RefZ–cyt-SpoIIE complex is formed. The observed tetrameric and oligomeric forms of cyt-SpoIIE found in complex with RefZ are in good agreement with the same forms of SpoIIE previously detected in live sporulating cells determined by Slimfield microscopy (36). A comparison of the RefZ-cyt-SpoIIE elution profile with that of cyt-SpoIIE alone shows that an increased fraction of cyt-SpoIIE was present in the oligomeric form, suggesting that RefZ could either promote SpoIIE oligomerization or stabilize SpoIIE multimers. Taken together, these results confirmed the formation of a RefZ–cyt-SpoIIE protein complex.

### Localization of RefZ during asymmetric septum formation

Previous localization experiments found that RefZ-GFP produced from its native promoter was detectable after 1 h of sporulation, when the axial filament was formed, and that it was localized diffusely and in weak foci near the cell poles (29). Around the time of polar division, RefZ-GFP was observed near the midcell at or near the cell membrane. Polar foci and diffuse signals were still detected at this time in some cells (29).

**Figure 4:**
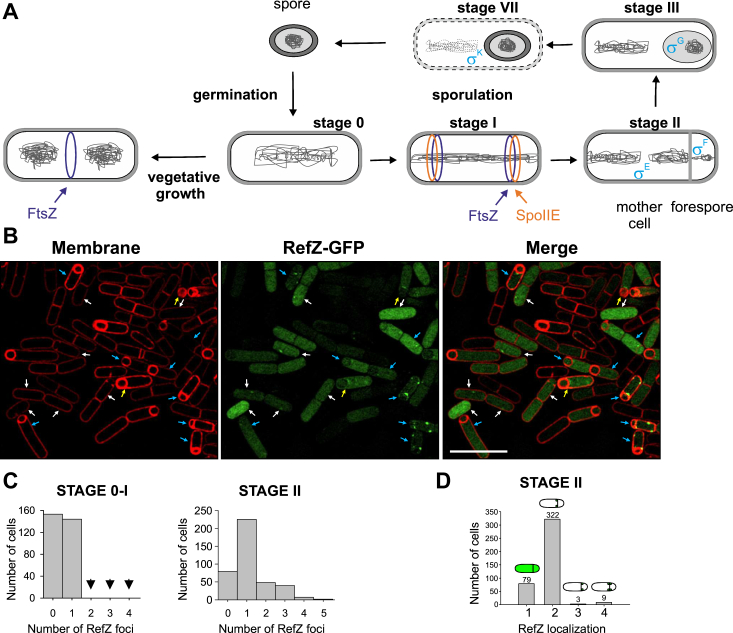
**Localization of RefZ and SpoIIE.***A*, vegetative growth, sporulation, and germination are illustrated. The midcell and asymmetric cell divisions during life cycle of *B*acillus *subtilis* are shown during the early stages (I-III) and the last stage (VII) of sporulation. The formation of FtsZ (Z-ring) is depicted in *blue* and the formation of SpoIIE (E-ring) is shown in *orange*. The activated compartment-specific sigma factors are shown in *cyan*. *B*, localization of RefZ during different stages of sporulation as observed by SIM. A sporulating culture of strain IB1820 (RefZ-mGFP) was observed by SIM. RefZ-mGFP signals are shown in *green*; the membranes were visualized using FM4-64 and are shown in *red*. *Arrows* in the SIM images indicate cells in different stages: *white*–stage 0–I, *blue*–stage II and *yellow*–stage III and later. The scale bars represent 5 μm. *C* and *D*, RefZ localization in the cells, in different stages of sporulation. A total of 297 cells (from three independent replicates – ∼100 cells per replica) from stages 0–I and 413 cells (from three independent replicates – ∼135 cells per replica) from stage II were analyzed for RefZ localization. Panel (*C*) shows a diagram of the numbers of RefZ foci in stages 0-I and stage II. Panel (*D*) shows a diagram of compartment-specific localization of RefZ in cells in stage II when the asymmetric septum is formed. RefZ localization is classified into the following groups: (1) diffused signal in the entire cell, (2) one or two foci at the mother cell side of the polar septum, (3) one or two foci at the forespore side of the septum, and (4) foci at both sides of the septum. Total numbers of cells and schematic cell drawings are shown above each column. SIM, structured illumination microscopy.

To follow the RefZ localization in more detail and to provide a basis for colocalization studies with SpoIIE, we prepared several strains in which RefZ at its native locus was fused to sequences encoding either mGFP (IB1820) or mScarlet (IB1821). The fusions were expressed under the control of the native promoter. Sporulation efficiency of these strains determined as heat-resistance was like WT (Table S1). Immunoblot analysis of IB1820 cells that were induced to sporulate and harvested at the third hour of sporulation revealed that RefZ-mGFP was produced and no degradation products were observed (Fig. S2). Using structured illumination microscopy (SIM), we statistically analyzed the localization and foci of RefZ-mGFP during the first hours of sporulation (Fig. 4, *B*–*D*). At early time points (stages 0–I), we observed both diffused signal and a diffused signal with one RefZ-mGFP focus at the midcell site or near the cell poles (Fig. 4*B*, C-left hand diagram). This is consistent with the previously reported localization pattern of RefZ during stage I of sporulation (29). During stage II of sporulation, RefZ is localized in the cells in four different ways. Most of the cells have RefZ foci at the mother cell side of the polar septum (Fig. 4, *B* and *D*). In some other cases, we detected RefZ as diffused signal in the entire cells and only in a few cells the RefZ foci are localized from the forespore side of the septum or at both sides of the septum (Fig. 4, *B* and *D*).

### RefZ and SpoIIE localize at the site of asymmetric septum formation

**Figure 5:**
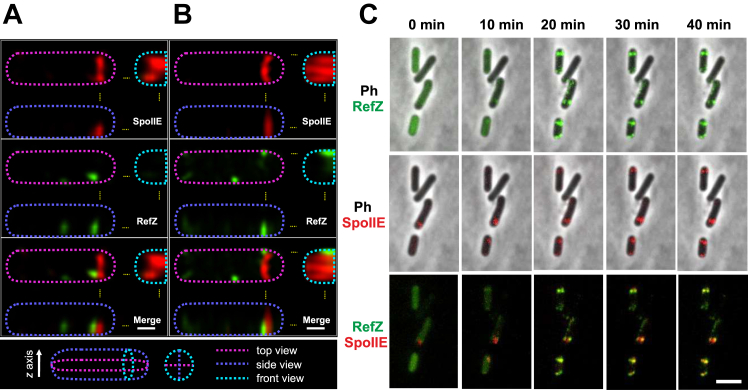
**Localization of RefZ and SpoIIE at the site of asymmetric septum formation.***A* and *B*, localization of RefZ and SpoIIE in a culture of strain IB1823 (SpoIIE-mScarlet, RefZ-mGFP) observed by SIM. Columns (*A*) and (*B*) are two representative cells in two different stages of sporulation, (*A*) the cell is in stage I; (*B*) the cell in stage IIii. In each panel, *dashed lines* indicate cell boundaries that were determined by increasing the intensity of the fluorescence signal. *Pink*, *blue*, and *cyan dashed lines* represent slices of cell area from different views as shown in the legend below the image. *Yellow dashed lines* indicate sites where slices were taken. The scale bar represents 0.4 μm. *C*, time-lapse localization of SpoIIE-mScarlet (*red*) and RefZ-mGFP (*green*) in a sporulating culture of strain IB1823 observed by fluorescence microscopy. Images in the first row are overlay of phase contrast with GFP fluorescence; images in the second row are phase contrast overlay with Scarlet fluorescence; images in the third row are an overlay of GFP and Scarlet. The scale bar represents 3 μm. SIM, structured illumination microscopy.

To follow the localization of RefZ and SpoIIE simultaneously, we prepared strain IB1823 expressing RefZ-mGFP and SpoIIE fused to mScarlet; both fusions were produced from their respective native promoters. SIM experiments revealed that RefZ localized as foci at the asymmetric septum close to the cell membrane and preferentially from the mother cell side of the E-ring (Fig. 5, *A* and *B*). Time-lapse localization of RefZ and SpoIIE showed that both proteins assembled as bright spots at the site of septation at the same or similar times (Fig. 5*C* and Video S1). The colocalization of these proteins at the asymmetric septum indicates that RefZ and SpoIIE might cooperate in determining the septation site.

### Localization of RefZ is dependent on SpoIIE

**Figure 6:**
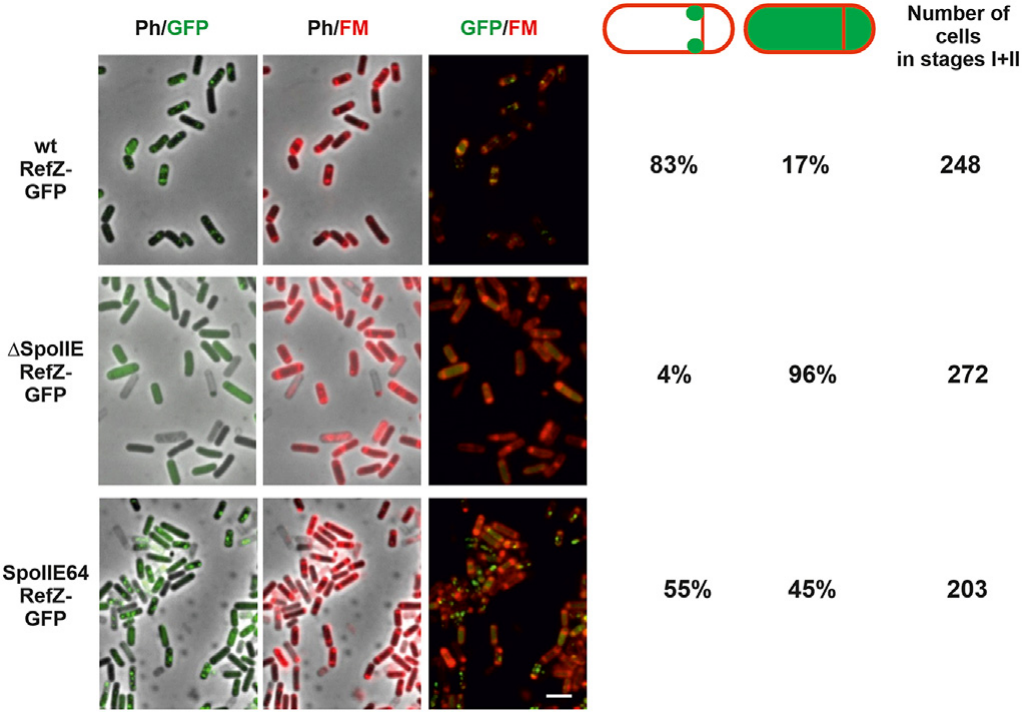
**Localization of RefZ to the polar septum requires SpoIIE.** Images in the first column: overlay of phase contrast with GFP fluorescence; images in the second column: overlay of phase contrast with membranes visualized using FM4-64; images in the third column: overlay of GFP and membranes. The scale bar represents 3 μm. The *right* panel shows the fraction of cells in stages I and II in which RefZ-mGFP was at a polar septum or diffused in the whole cells. The right column shows the total number of evaluated cells in stages I and II.

Since these experiments show that SpoIIE likely interacts with RefZ at the polar septum, we asked whether the absence of SpoIIE could influence the localization of RefZ. To test this, we prepared a *spoIIE* deletion strain, IB1824, in which RefZ-mGFP is expressed under the control of its promoter. We observed that in 96% of Δ*spoIIE* cells in sporulation stage II, the RefZ-mGFP signal was dispersed throughout the whole cell compared to only 17% for the WT (Fig. 6). In both genetic backgrounds, the RefZ-mGFP signal was also detected at the membrane, indicating that RefZ can still interact there with other membrane proteins. Western blot analysis of RefZ-mGFP revealed that no significant degradation occurred in Δ*spoIIE* cells (Fig. S2).

To further analyze the effect of SpoIIE on RefZ localization, we prepared strain IB1825 where RefZ-mGFP was expressed in the *spoIIE64* phosphatase activity deficient strain, which contains a single missense substitution (L646F) in the C-terminal phosphatase domain of SpoIIE (16). This mutant is abortively disporic with the majority of cells assembling thin septa at one or both the polar positions. We observed that in 45% of cells, the RefZ-mGFP signal was dispersed, indicating that SpoIIE targets RefZ to the septum, but as this interaction is transient and sporulation does not proceed further in this mutant, RefZ is released from the septum (Fig. 6).

### SpoIIE influences cell division when expressed during vegetative growth

**Figure 7:**
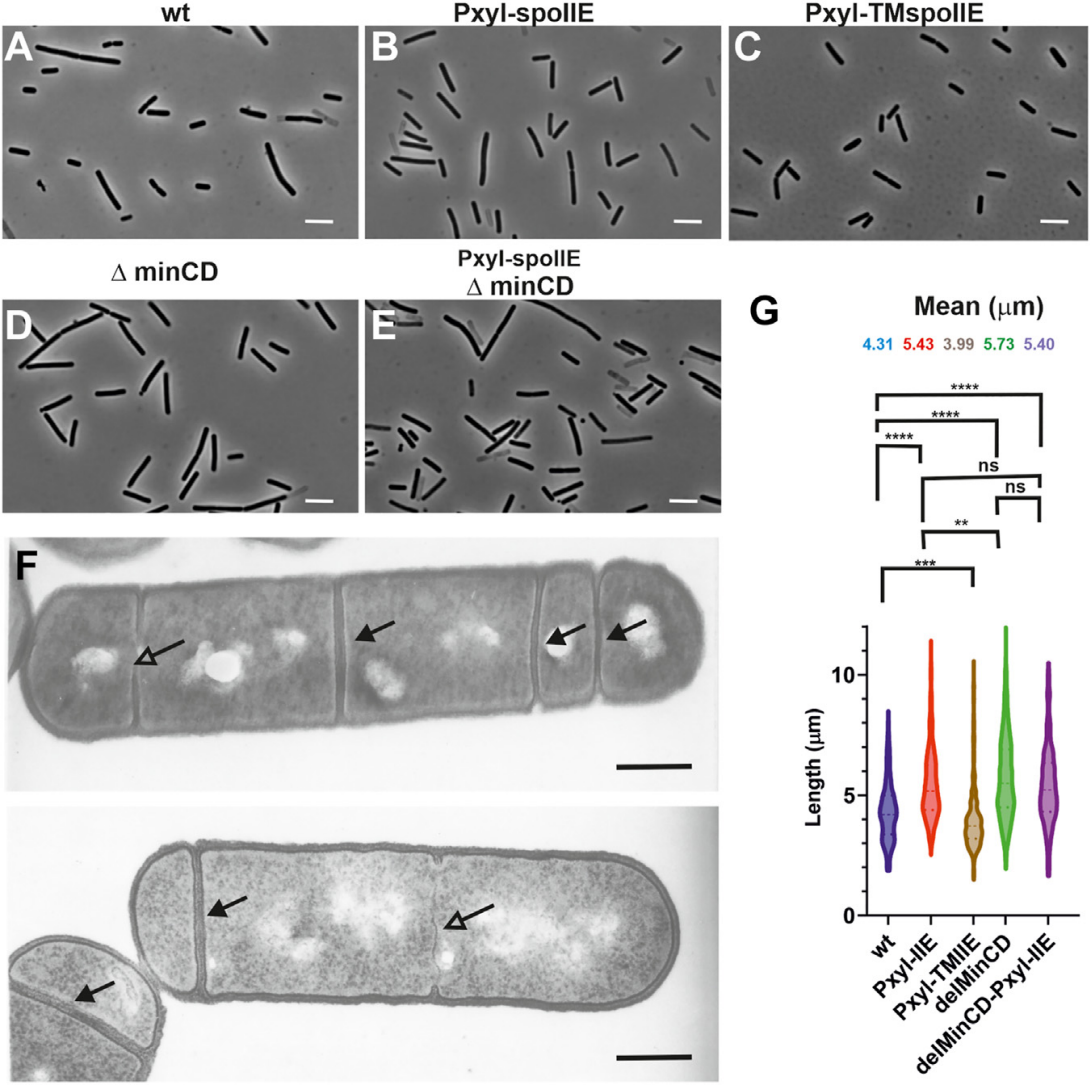
**Expression of SpoIIE during vegetative growth increases cell length.** Representative phase contrast images of strains (*A*) WT - PY79, (*B*) *Pxyl-spoIIE* – IB1831, (*C*) *Pxyl-TMspoIIE* – IB1832, (*D*) Δ*minCD* – IB1371, (*E*) *Pxyl-spoIIE*, Δ*minCD* – IB1833. The scale bar represents 4 μm. *F*, TEM images of strains during exponential growth in which the expression of SpoIIE was induced with 0.5% xylose. The scale bar represents 0.6 μm. *Arrows* point to thick vegetative-like septa; open arrows point to ultrathin septa. *G*, violin plot of cell length distribution for the indicated strains. More than 400 cells were analyzed for each sample. Selected significant *p*-values from two-tailed unpaired *t*-tests are depicted above the plot; the threshold for statistical significance was taken to be *p* < 0.05. *Stars* indicate statistically significantly different values, where ns (not significant) corresponds to *p*-value > 0.05, ∗ to *p* ≤ 0.05, ∗∗ to *p* ≤ 0.01, ∗∗∗ to *p* ≤ 0.001 and ∗∗∗∗ to *p* ≤ 0.0001 g. TEM, transmission electron microscopy.

Finally, given the interaction of SpoIIE with the negative regulator of cell division RefZ and DivIVA during sporulation, we asked what would happen if SpoIIE was artificially produced during vegetative growth. To answer this question, we prepared a strain expressing *spoIIE* fused with *ypet* under the control of a xylose-inducible promoter (IB1831). We then induced the expression of SpoIIE-YPet, determined its amount, and measured the cell length after 2.5 h of growth (Fig. 7, *A*–*G* and Fig. S3). Interaction of SpoIIE with negative regulators during vegetative growth should lead to cell elongation. As expected, we found that expression of SpoIIE-YPet during vegetative growth did indeed lead to cell elongation (cell length was 5.43 μm in comparison to 4.31 μm for WT cells), indicating cell division inhibition by SpoIIE (Fig. 7*G*). This early induction of SpoIIE decreased the sporulation efficiency of this strain (Table S1).

To determine which negative regulators cause this effect, we prepared a strain expressing *spoIIE* fused with *ypet* under the control of a xylose-inducible promoter in Δ*minCD* background (IB1833). We expected to find that the absence of the negative regulator MinC would lead to a reduction in cell length. Surprisingly, this was not the case: the expression of SpoIIE-YPet during vegetative growth in the absence of MinCD had no obvious effect on cell length [(5.4 μm *versus* 5.43 μm for the SpoIIE-expressing WT strain) (Fig. 7*G*)]. To summarize, cell lengthening probably occurs in a MinCD-independent manner.

To analyze the elongation phenotype of cells expressing SpoIIE during vegetative growth, we examined thin sections of cells using electron microscopy to check the morphology of the septa. These experiments revealed the presence of multiple septa in the cells (Fig. 7*F*). Based on these results, we propose that the presence of SpoIIE is compatible with septum formation during vegetative growth and that it can lead to the formation of multiple septa in some cells. We also observed “ultrathin” septa with a width as little as 10 nm (Fig. 7*F*) in comparison to the normal WT vegetative septum with a width of 80 nm or the WT sporulation septum with a width of 25 nm (24). Taken together, SpoIIE as a positive regulator of septation can initiate multiple and thin septa formation. Therefore the cell cannot divide at these sites, which leads to the observed cell elongation.

Transmembrane proteins can sometimes cause cell elongation regardless of the particular function of the protein they belong to (37). To verify that the cell elongation we observed was not due simply to the increased number of copies of the SpoIIE transmembrane domain alone, we constructed a control strain IB1832 expressing only transmembrane helices of *spoIIE*. The results showed that these cells are even shorter than the WT cells [(3.99 μm in comparison to 4.31 μm of WT control) (Fig. 7*G*)].

## Discussion

In this study, we identified a new binding partner of SpoIIE, the RefZ protein, and provided evidence about the SpoIIE-RefZ spatio-temporal interplay during the process of asymmetric septum formation. Using a BACTH system, we showed that SpoIIE interacts with the negative regulator of cell division RefZ. More detailed analyses then revealed that the central regulatory domain of SpoIIE is crucial for interaction with the cytosolic RefZ protein. The interaction between SpoIIE and RefZ was further confirmed by pull-down and gel filtration assays.

We characterized the spatio-temporal localization of these proteins during the early stages of sporulation. We observed that RefZ colocalized with SpoIIE at the polar septum in stage II, where RefZ formed foci preferentially from the mother cell side of the septum. This RefZ localization was dependent on SpoIIE since the majority of the RefZ-mGFP signal in this stage was dispersed throughout the entire cell in a Δ*spoIIE* mutant.

**Figure 8:**
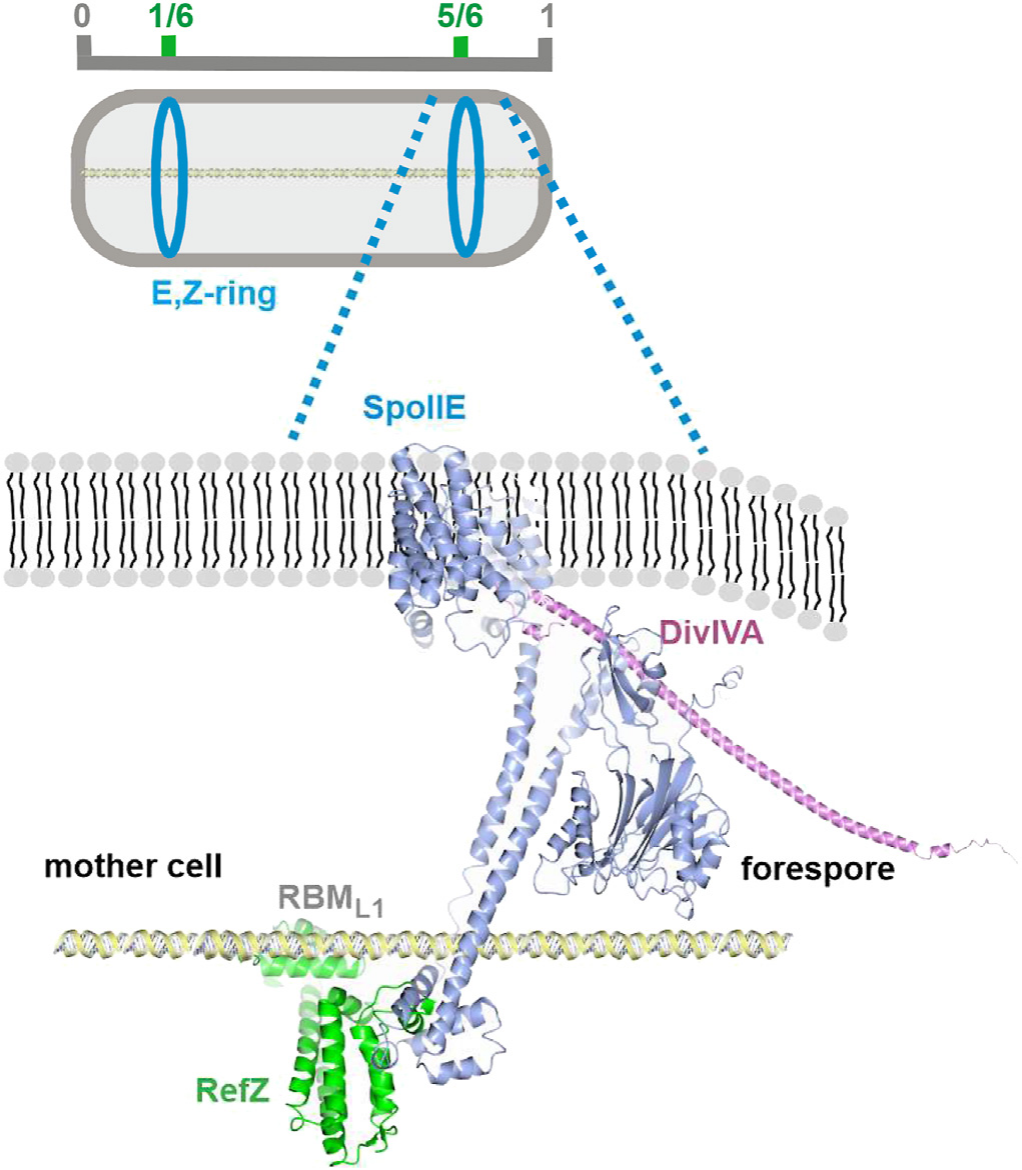
**Simplified model of sporulation septum site determination.** The model shows how the precise site of the asymmetric septum at one-sixth of the cell length can be recognized by positive and negative regulators at stage I of sporulation. At this site, the Z-ring (not shown in the model *below*) and E-ring can be formed. RefZ binds to specific DNA sequences, *R*efZ *b*inding *m*otifs (RBM), on both chromosomes, stretched from one pole to other as an axial filament. RBM sequences are located close to the *ori* regions of the two chromosomes. In the model shown, RBM_L1_ is depicted where RefZ binds through its helix-turn-helix domain. The 3D crystal structure of RefZ (35) is depicted in *green*. The 3D AlphaFold model of DivIVA (https://www.uniprot.org/uniprotkb/P71021/) is depicted in *violet* and the AlphaFold model of SpoIIE (https://www.uniprot.org/uniprotkb/P37475/) is in *blue*. The 3D structures are simply superimposed as the interaction interfaces of the proteins are unknown. For simplicity, only one molecule of each studied protein is shown and structures of additional proteins present in the early division complex, such as FtsA, SepF or MinJ, MinCD are omitted.

The selection of the sporulation septum site at a precisely determined asymmetric position might be essential for at least two reasons. First, it could be that a specific volume ratio between the mother cell and the forespore is required for σ^F^ to be properly activated only in the forespore (20). Second, it could be that the size of the spore needs to be big enough to store the chromosome but also small enough to be effectively encapsulated by the spore coat (38). As mentioned above, soon after the cell enters sporulation and before the sporulation septum begins to form, the chromosomes significantly change their morphology and form an axial filament (4). The asymmetric septum then bisects this axial filament at the precise site to ensure correct chromosome trapping in the forespore (39) (Fig. 4*A*). As RefZ specifically binds to DNA (30, 35) and, as we showed here, interacts with SpoIIE, we suggest that the transient SpoIIE-RefZ-DNA complex may dictate the position of the Z-ring at this proper septation site relative to the chromosome. We hypothesize that the measuring device that is used by the cell to find the asymmetric septum site is the nucleoid itself. In this device, one of the *ori* sequences is attached to one pole and represents 0 on this “measuring tape” (Fig. 8). The second *ori* sequence, attached to the opposite pole, represents 1. Some of the RefZ-specific binding sequences on the two nucleoids are at one-sixth and five-sixths of the cell length, helping to determine the sporulation septum site. The absence of RefZ then moves the sporulation septum toward the midcell (25). Interestingly, cells lacking RefZ sporulate to near-WT levels (29). In the opposite direction (that is, toward the pole), the sporulation septum site determination may be mediated by the Min system since the deletion of the Min system causes the asymmetric septum to move closer to the nearest pole (25). In agreement with this, it was observed recently that DivIVA, as a part of Min system, localizes preferentially from the forespore side of the asymmetric septum (27, 40). It is also known that while the absence of DivIVA significantly decreases sporulation efficiency (28), it was shown recently that the deletion of *minCD* caused a mild sporulation defect (40). The absence of MinJ was initially reported to decrease sporulation efficiency to 36% compared to the WT (41), which is in contrast with a new study (40). All these data indicate that the role of the individual contributions of these Min system proteins in sporulation remains elusive. Nevertheless, we speculate that the interaction of SpoIIE with DivIVA as a part of Min system can serve as an obstacle to the movement of the Z- and E-rings closer to the cell pole, effectively providing a backstop. Taken together, we hypothesize that SpoIIE mainly has a positive role in septum formation and that the transient interaction of the negative regulators of cell division with SpoIIE could serve to fine-tune the localization of the proper sporulation septation site. Importantly, the localization of RefZ as well as the Min system proteins are determined by the precise spatial organization of the nucleoid and its interaction with SpoIIE marks the cell division site in the membrane. However, it is important to point out that very likely other proteins are involved in these early stages of asymmetric cell division complex formation. Nevertheless, further experiments are required to elucidate in more detail how this complex mechanism works.

Considering the important positive role of SpoIIE in asymmetric septum formation and its interactions with Min system as the negative regulator of cell division, we also examined the effect of SpoIIE expression on cell division during vegetative growth. We found that the expression of SpoIIE during vegetative growth leads to cell elongation. However, this does not likely arise from its interaction with the negative regulator, Min system, since their absence does not restore the WT cell length. Electron microscopy images of vegetative cells expressing the entire SpoIIE revealed the presence of several septa of different thicknesses, even “ultrathin” septa. Very recently, it was shown, that SpoIIE can regulate the thickness of the sporulation septum (24). The presence of septa with different thicknesses in our case could be due to the nonphysiological expression of SpoIIE from an inducible promoter at an improper time, *i.e.* during vegetative growth. As SpoIIE interacts directly with GpsB and RodZ, which are important for interconnection with the peptidoglycan synthesizing machinery (32, 34, 42), we can speculate that various amounts of peptidoglycan could be inserted into these septa. Taken together, we propose that SpoIIE can positively affect septum formation when expressed during vegetative growth probably through interactions with FtsZ and other division proteins. However, the septa can be thinner than a normal vegetative septum and thus these cells cannot divide and become elongated.

Finally, and importantly, SpoIIE is universally conserved and indispensable for sporulation septum formation in endospore formers such as *Bacilli* and *Clostridia* (43). The necessity of SpoIIE for sporulation stems from its functions that are associated with the formation and structure of the asymmetric septum, the activation of σ^F^ and peptidoglycan remodeling during forespore engulfment. The second component of this mechanism, RefZ and its binding sites on the chromosome (RBMs) are conserved across the *Bacillus* genus (30). Additionally, as we showed previously, *Bacilli* and *Clostridia* possess homologs of the *B. subtilis* Min system proteins (44) indicating the conservation of the third component of this mechanism. However, some *Clostridia* species do have different combinations of Min proteins and also include a MinE homolog, which allows oscillation of the *Clostridium difficile* Min system when transplanted into *B. subtilis* (44). Interestingly, the sporulation of those strains exhibiting oscillation was diminished, indicating that oscillation interferes with sporulation in *B. subtilis.* Collectively, the presence and conservation of these key proteins in endospore-forming bacteria indicate that the mechanism by which the asymmetric division site is determined is likely to be conserved.

In conclusion, our study shows direct contact between the membrane protein, SpoIIE and DNA-binding protein, RefZ during sporulation. This interaction is transient and manifests during the early stages of asymmetric septum formation. Our results point out the important role of these proteins in the determination of the site where the sporulation septum is formed. It is highly plausible that additional proteins are involved in the development of this initial phase of the asymmetric cell division complex. Further research is required to completely understand the role of each protein involved in controlling this event.

## Experimental procedures

### Bacterial strains and plasmids

*E. coli* strains were grown in LB (45), *B. subtilis cells* were grown in LB or Difco sporulation medium (46). When required, media were supplemented with 10 μg/ml kanamycin, 5 μg/ml chloramphenicol, 100 μg/ml spectinomycin, 1 μg/ml erythromycin, and 25 μg/ml lincomycin. p_xyl_-driven expression was induced using 0 to 0.5% xylose. In general, all molecular biology experiments in *B. subtilis* were carried out as described previously (46).

The bacterial strains used in this study are listed in Table S2; the prepared *B. subtilis* strains are derivatives of *B. subtilis* PY79 (47); *E. coli* strains MM294 (48); and DH5α (Invitrogen) were used for cloning and plasmid isolation. The plasmids used in this study are listed in Table S3; the sequences of the oligonucleotides used in this work are given in Table S4.

pSGrefZ-mscarlet was constructed in two steps. First, *mscarlet* was PCR amplified from expression vector pET14 (b+) mscarlet (kind gift from Mark Leake) using the primers mscarletSKpn and mscarletEPst and ligated into a pSG1151 vector (49), resulting in pSGmscarlet. A 273 bp fragment containing the C-terminal part of *refZ* (117–207 aa) was amplified using refZSKpn and refZEKpn primers, digested with KpnI and cloned into the KpnI site of pSGmscarlet to create the integration plasmid pSGrefZ-mscarlet. The integration plasmid pSGIIE-mscarlet was prepared by using pSGmscarlet and a KpnI fragment from pSGIIE-YPet encoding the C terminus of SpoIIE (724–827 aa) (34). To construct pUCkanrefZ-mgfp, we took advantage of the recombinant plasmids pUCkan and pSGmgfp prepared for other studies (32, 36). pSGmgfp was digested with BamHI and KpnI and the resulting 713 bp fragment containing *mgfp* was ligated into a similarly cut pUCkan vector. Subsequently, the 273 bp KpnI fragment containing the C-terminal part of *refZ* obtained from the KpnI digestion of pSGrefZ-mscarlet was ligated into the KpnI digested pUCkanmgfp plasmid to create the pUCkanrefZ-mgfp integration vector.

To study the effect of SpoIIE expression during vegetative growth, pSG54IIE-YPet and pSG5410TMIIE-YPet were prepared. First, *ypet* was PCR amplified from pSGIIE-YPet using the primers ypetSK and ypetESpe, digested with KpnI and SpeI, and ligated into similarly cut pSG1154 (49) to yield pSG54YPet. To construct pSG54IIE-YPet, the entire *spoIIE* gene (2484 bp) was amplified by using spoIIESK and spoIIEEK primers, digested with KpnI and cloned into the KpnI site of pSG54YPet to give pSG54IIE-YPet. pSG5410TMIIE-YPet was constructed similarly; the transmembrane region of *spoIIE* (993 bp) was amplified by using spoIIESK and spoIIE10TMEK primers, digested and cloned, into pSG54YPet to give pSG5410TMIIE-YPet.

To analyze the interaction between SpoIIE and RefZ using a pull-down assay, we used a previously prepared pETspoIIE-S, which allows the expression of the S-tagged cytosolic part of SpoIIE (34), and we created pETrefZ, which allows the expression of His-tagged RefZ. To construct pETrefZ, a PCR fragment containing *refZ* was amplified using the RefZSB Eve and RefZEE Eve primers and, after digestion with BamHI and EcoRI, was cloned into a similarly digested pETDuet-1 vector. To analyze the formation of the SpoIIE–RefZ complex by gel filtration, we used pTAR-spoIIE B2 (375–827 aa), which expresses the His-tagged cytosolic part of SpoIIE (14), and pETrefZ, which expresses His-RefZ.

### Bacterial two-hybrid system and quantitative β-galactosidase assay

*refZ* (624 bp) was PCR amplified using a combination of primers refZSB and refZEE or refZSB and refZ25EE, respectively. PCR fragments were digested with BamHI and EcoRI and cloned into the vectors of a BACTH bacterial two-hybrid system (31) to generate plasmids encoding RefZ fused to the T25 and T18 fragments of adenylate cyclase. Plasmids encoding SpoIIE or SpoIIE domains were described previously (34); plasmids encoding DivIVA were a kind gift from R. Daniel. To test for protein-protein interactions, each pair of plasmids was cotransformed into *E. coli* BTH101. Cotransformation mixtures were spotted onto LB plates supplemented with 40 μg/ml X-Gal (5-bromo-4-chloro-3-indolyl-β-d-galactopyranoside), 0.5 mM IPTG, 100 μg/ml ampicillin, and 30 μg/ml kanamycin, and grown for 24 to 48 h at 30 °C. β-galactosidase activity was measured as described by Miller (50) with an extra wash step.

### Protein isolation and purification

*E*. *coli* BL21 (DE3) strains harboring expression plasmids, pETspoIIE-S or pETrefZ, respectively, were grown in LB medium at 37 °C. When the *A*_600_ of the culture reached 0.5, the expression of recombinant proteins was induced by the addition of 1 mM IPTG. After 3 h of further growth at 37 °C, or overnight growth at 16 °C in the case of cyt-SpoIIE, the cells were harvested by centrifugation. Cell pellets from 50 ml of cultures of His-RefZ, and cell pellets from 150 ml of cyt-SpoIIE-S, respectively, were resuspended in the lysis buffer (20 mM Tris–HCl pH 8.5, 150 mM NaCl, and 1 mM 4-(2-aminoethyl) benzenesulfonyl fluoride [AEBSF]) and combined (cyt-SpoIIE-S+His-RefZ) before being lysed by sonication. The lysates were centrifuged at 30,000 rpm for 30 min to remove cell debris and loaded onto 1 ml Ni Sepharose HP columns (GE HealthCare). After thorough washing with buffer containing 40 mM imidazole, proteins were eluted with 1 M imidazole. Coeluted proteins were identified by Western blot analysis using monoclonal antibodies against the His-tag (cat. number 70796-3, Lot: 3172550, Novagen) or the S-tag (cat. number 71549-3, Lot: 3172550, Novagen).

### Gel-filtration chromatography assay

Cyt-SpoIIE comprising 375 to 827 aa was isolated largely as described previously (14). Briefly, the cells were lysed by sonication, the suspension was clarified by centrifugation (30,000 rpm for 30 min) and the extracts were fractionated by metal chelation chromatography on a Ni Sepharose HP column (GE HealthCare). cyt-SpoIIE was highly enriched in fractions eluting from this column at 200 mM imidazole. RefZ was isolated in principle as described above except that an imidazole step gradient was used for elution; RefZ was mainly eluted at 300 mM imidazole. Gel-filtration chromatography was performed on a Superdex S200 16/60 gel filtration column (GE HealthCare) equilibrated in 50 mM Tris–HCl pH 8.5, 300 mM NaCl buffer. To analyze the formation of RefZ-cyt-SpoIIE complex 350 μl of RefZ (28.8 μM) was premixed with 350 μl of cyt-SpoIIE (15.5 μM), dialyzed overnight and 500 μl of this mixture was loaded onto gel filtration column. Fractions were analyzed by Western blotting using an anti-His monoclonal antibody (cat. number 70796-3, Lot: 3172550, Novagen). The following molecular weight standards were used for column calibration: thyroglobulin (669 kDa), ferritin (440 kDa), catalase (232 kDa), aldolase (158 kDa), bovine serum albumin (67 kDa), ovalbumin (43 kDa), chymotrypsinogen A (25 kDa), and ribonuclease A (13.7 kDa). Blue dextran (∼2000 kDa) was used to determine the void volume of the column.

### Fluorescence microscopy and image acquisition

A single colony of *B. subtilis* strain was resuspended in 100 μl of LB medium, spread onto LB plate and grown overnight at 30 °C. The overnight culture was subsequently used to inoculate 10 ml of Difco sporulation medium to an *A*_600_ of 0.1. The culture was then grown at 37 °C and cells were harvested 2 and 3 h after the onset of stationary phase. For membrane visualization, the fluorescent dye fluorescence microscopy 4 to 64 (Molecular Probes) was used at concentrations of 0.2 to 1 μg/ml. Samples were stained with 0.2 μg/ml 4′,6-diamidino-2-phenylindole to visualize DNA. Cells were examined under the microscope on 1% agarose covered slides. When it was necessary to increase the cell density, cells were concentrated by centrifugation (3 min at 2500 rpm) and resuspended in a small volume of supernatant before microscopic examination. All images were obtained with an Olympus BX63 microscope equipped with a sCMOS Zyla-4.2P camera (Andor). Olympus CellP imaging software (https://www.olympus-lifescience.com/en/software/cellsens/) and ImageJ/Fiji (https://fiji.sc/) were used for image acquisition and analysis.

### SIM experiments

Bacteria were grown in the same way as for conventional fluorescence microscopy (see above). One milliliter of sporulating culture was pelleted (3 min, 2500 rpm) and resuspended in 100 μl of 1 × PBS. One microliter of the sample was spotted on a coverslip and covered with a thin agarose pad (1.5% agarose in 1 × PBS). The samples were observed using a DeltaVision OMX equipped with a 60 × 1.42, PlanApo N, oil immersion objective and softWoRx Imaging Workstation software (https://download.cytivalifesciences.com/cellanalysis/download_data/softWoRx/7.2.1/SoftWoRx.htm). GFP-tagged proteins were imaged using a 488 nm excitation laser; Nile Red stained membranes and proteins tagged with mScarlet were imaged using a 568 nm excitation laser. For membrane staining, Nile Red was added to 1 ml of sporulating culture at a final concentration of 10  μg/ml. After 10 min of incubation at room temperature, bacteria were pelleted and washed once with 1 × PBS. If needed, DNA was stained with 0.1 μg/ml 4′,6-diamidino-2-phenylindole followed by washing in 1 × PBS. We imaged >100 cells for each studied strain. ImageJ/Fiji (https://fiji.sc/) and Imaris (https://imaris.oxinst.com/) were used for image processing.

### Transmission electron microscopy

Strain IB1831 was grown in LB medium supplemented with 0.5% xylose. Samples for transmission electron microscopy were harvested by centrifugation during the logarithmic cell growth phase (around 2 h after inoculation), washed in a buffer containing 0.1 M Na cacodylate (pH 7.2), and resuspended for fixation in the same buffer containing 2% glutaraldehyde and 1% OsO_4_ (23). Cells were fixed by incubation for 1 h at 48 °C. Following fixation, cells were pelleted by centrifugation, washed in 0.1 M cacodylate buffer, and dehydrated by a series of washes and incubations (10 min each) in graded concentrations of ethanol (30, 50, 70, 85, 95, and 100% [twice in 100% ethanol]). This was followed by washing (twice for 30 min each) in acetone. Dehydrated cells were embedded in Spurr medium and polymerized for 8 h at 70 °C. Samples were sectioned (40- to 120-nm thickness) on a NOVA3 ultramicrotome (LKB) and floated onto copper grids. Sections were stained on uranyl acetate drops for 20 min and on lead citrate drops for 5 min. Stained thin sections were examined and photographed on Tesla Brno (T541) or JOEL-100 CX electron microscopes.

## Data availability

Original microscopic images are available upon request. All data generated or analyzed during this study are included in this published article and its supplementary files, or available upon request. Source data are provided with this article.

## Supporting information

This article contains supporting information (16, 23, 31, 34, 47, 48, 49, 51, 52, 53).

## Conflict of interest

The authors declare that they have no conflicts of interest with the contents of this article.
