# Supplementary material for: Spatio-temporal control of asymmetric septum positioning during sporulation in Bacillus subtilis
Source: J Biol Chem. 2024 May 4;300(6):107339. doi: 10.1016/j.jbc.2024.107339 (PMC11154705; doi:10.1016/j.jbc.2024.107339)
Supplement: Supporting Tables and Figures [file mmc2.pdf]

Supporting Information

Spatio-temporal control of asymmetric septum positioning during sporulation in *Bacillus subtilis*

Katarína Muchová, Jiří Pospíšil, Evelína Kalocsaiová, Zuzana Chromiková, Silvia Žarnovičanová, Hana Šanderová, Libor Krásný and Imrich Barák

Table S1. Sporulation efficiency of IB1820, IB1821 and IB1831 determined as heat resistance and reported as relative to wild type.

strain	relevant genotype	sporulation efficiency (%)
PY79	wild type	100
IB1820	p_{refZ}-*refZmgfp*	96±1.5
IB1821	p_{refZ}-*refZmscarlet*	97±2.5
IB1831	*amyE::*p_{xyl}-*spoIIEypet*	57±2.3

Table S2. Bacterial strains

Strain	Genotype	Source/reference
B. subtilis		
PY79	Prototrophic derivative of *B. subtilis* 168	47
PY180	*spoIIE* ::Tn*917* Ω*HU7*	23
PY507	*spoIIE64 trpC2*	16
IB1820	p_{refZ} *refZ-mgfp kan*	this work
IB1821	p_{refZ} *refZ-mscarlet cat*	this work
IB1822	p_{spoIIE} *-spoIIE-mscarlet- cat*	this work
IB1823	p_{refZ} *refZ-mgfp kan* p_{spoIIE} *-spoIIE-mscarlet- cat*	this work
IB1824	p_{refZ} *refZ-mgfp kan spoIIE::Tn917ΩHU7*	this work
IB1825	p_{refZ} *refZ-mgfp kan spoIIE64 trpC2*	this work
IB1831	*amyE::*p_{xyl} *-spoIIE-ypet sp*	this work
IB1832	*amyE::*p_{xyl} *-10TMspoIIE-ypet sp*	this work
IB1371	*minCD::kan*	51
IB1833	*amyE::*p_{xyl} *-spoIIE-ypet sp minCD::kan*	this work
MM294	F^- *endA-1 hsdR-1, (*rk^-*,* mk^+*) supE44 thi-1 recA1*	48
DH5α	F^- *Φ80lacZΔM15 Δ(lacZYA-argF) U169 recA1 endA1 hsdR17 (*rK^-*,*	Invitrogen
BL21(DE3)	*hsdS gal (λcts857 indt Sam7 nin5 lacUV5-T7gene*	Novagen
BTH101	F^- *cya-99 araD139 galE15 galK16 rpsL1(*Str^r*)hsdR2 mcrA1 mcrB1*	31

Table S3. Plasmids

Plasmid	Description	Reference
pSG1151	*bla cat gfpmut1*	49
pSG1154	*bla spc gfpmut1*	49
pUK19	*bla kan*	52
pETduet-1	*expression vector used for proteins co-expression, bla lacI T7 promoter*	Novagen
pUC19	vector with a multiple cloning site (MCS) *bla*	53
pSGrefZ-mscarlet	*bla cat refZ (117-207aa)-mscarlet*	this work
pSGIIE-mscarlet	*bla cat spoIIE (724-827aa)-mscarlet*	this work
pUCkanrefZ-mgfp	*bla kan refZ (117-207aa)-mgfp*	this work
pSG54IIE-YPet	*bla sp* p_{xyl} *-spoIIE -ypet*	this work
pSG54TMIIE-YPet	*bla sp* p_{xyl} *-10TMIIE -ypet*	this work
pETrefZ	*bla lacI pT7refZ*	this work
pETIIE-S	*bla lacI pT7cyt-spoIIE-S*	34
pKT25	enable fusion to C-terminal end of adenylate cyclase T25 fragment	31
pKNT25	enable fusion to N-terminal end of adenylate cyclase T25 fragment	31
pUT18	enable fusion to N-terminal end of adenylate cyclase T18 fragment	31
pUTC18	enable fusion to C-terminal end of adenylate cyclase T18 fragment	31
pKTrefZ	p_{lac}*-T25-refZ kan*	this work
pKNTrefZ	p_{lac}*-refZ-T25 kan*	this work
pUTrefZ	p_{lac}*-refZ-T18 bla*	this work
pUTCrefZ	p_{lac}*-T18-refZ bla*	this work
pKTdivIVA	p_{lac}*-T25-divIVA kan*	R. Daniel
pKNTdivIVA	p_{lac}*-divIVA-T25 kan*	R. Daniel
pUTdivIVA	p_{lac}*-divIVA-T18 bla*	R. Daniel
pUTCdivIVA	p_{lac}*-T18-divIVA bla*	R. Daniel
pKTIIE	*plac-T25-spoIIE kan*	34
pKNTIIE	*plac-spoIIE-T25 kan*	34
pUTIIE	*plac-spoIIE-T18 bla*	34
pUTCIIE	*plac-T18-spoIIE bla*	34
pKTnctIIE	*plac-T25- spoIIE domain I,II kan*	34
pKNTnctIIE	*plac-spoIIE domain I,II-T25 kan*	34
pUTCnctIIE	*plac-T18-spoIIE domain I,II bla*	34
pUTnctIIE	*plac-spoIIE domain I,II T18 bla*	34
pKTctIIE	*plac-T25- spoIIE domain II kan*	34
pKNTctIIE	*plac-spoIIE domain II-T25 kan*	34
pUTCctIIE	*plac-T18-spoIIE domain II bla*	34
pUTctIIE	*plac-T18-spoIIE domain II bla*	34

Table S4. Oligonucleotides used in this work

Primer	Sequence 5′- 3′
refZSKpn	GAGCGAGACGGTACCGAAATTATGTCGACATATCTAATGAAG
refZEKpn	GAGCGAGGAGGTACCGTTGGTGAGCGCCACGTC
mscarletSKpn	GCGAACGCAGGTACCATGGTCTCCAAAGGAGAG
mscarletEPst	GCGAACGCACTGCAGTTATTTATACAGCTCATCCATAC
RefZSB Eve	GAAGGAGACGGATCCCATGAAAGTAAGCACCAAAGAC
RefZEE Eve	GGAGGGCCTGAATTCCTAGTTGGTGAGCGCCA
refZSB	GATGATGATGGATCCGATGAAAGTAAGCACCAAAGAC
refZEE	GATGATGATGAATTCGAGTTGGTGAGCGCCAC
refZ25EE	GATGATGATGAATTCGTTAGTTGGTGAGCGCCAC
ypetSK	GAGGCTGCGGGTACCATGTCTAAAGGTGAAGAATTATTC
ypetEspe	GAGGAGGACAACTAGTTTATTTGTACAATTCATTCATACC
spoIIESK	GAGGAGCAGGGTACCATGGAAAAAGCAGAAAGAAG
spoIIEEK	GAGGAGGCGGGTACCTGAAATTTCTTGTTTGTTTTGAAAG
spoIIE10TMEK	CACGAGGTGGGTACCATATCTCGCCACTTTCCTC

Figures

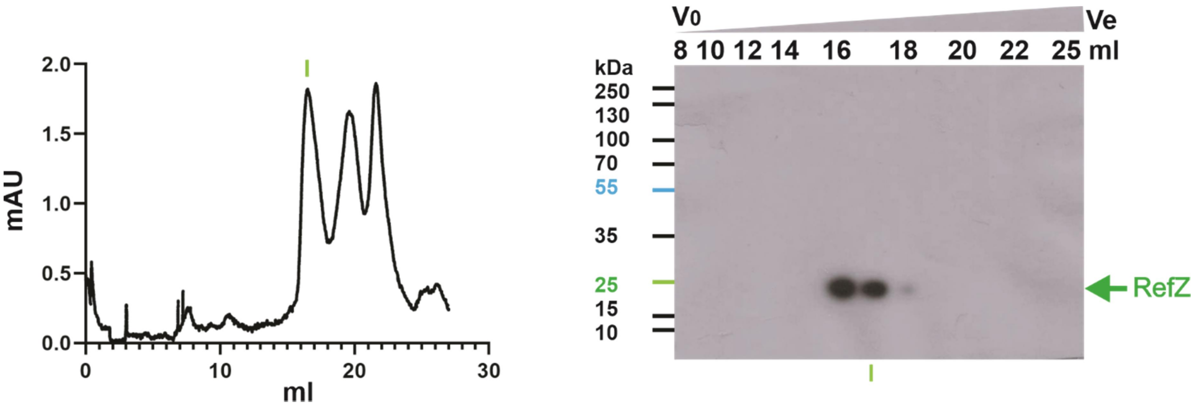

Figure S1. Western blot analysis of RefZ elution after gel filtration. RefZ was applied to the Superdex column and was eluted in a single peak (I in green) corresponding to a RefZ monomer as described in Fig. 3 legend. Chromatogram on the left is reused from Fig. 3A and in addition to Fig. 3A, fractions corresponding to two later peaks with elution volumes V_e = 19 ml and 22 ml are included in the right panel. Western blot shows no RefZ present in these peaks (right panel).

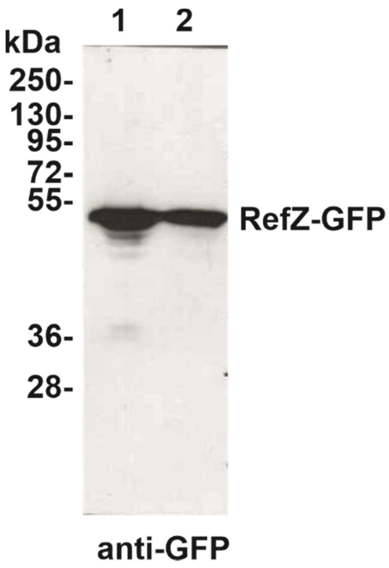

Figure S2. Western blot analysis of RefZ-mGFP in strains IB1820 and IB1824. The cells were induced to sporulate and harvested at the 3rd hour of sporulation. A monoclonal anti-GFP antibody (cat. number ab1218, Lot. GR213436-60, Abcam) was used for the detection of RefZ-mGFP. Lane 1 – RefZ-mGFP in a wild-type background (IB1820); lane 2 – RefZ-mGFP in *ΔspoIIE* (IB1824). Immunoblot analysis revealed that RefZ-mGFP was produced in both strains and no significant degradation occurred.

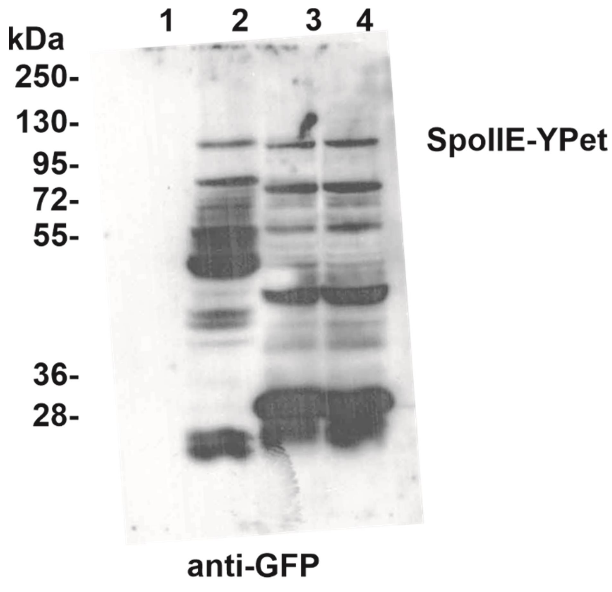

Figure S3. Western blot analysis of SpoIIE-YPet expression in strain IB1831 during vegetative growth. Cells were harvested after 2.5 hours of growth. A monoclonal anti-GFP antibody (cat. number ab1218, Lot. GR213436-60, Abcam) was used for the detection of SpoIIE-YPet. Lane 1 – wild-type (PY79); lane 2 – SpoIIE-YPet produced from its native promoter, when cells were harvested 2 hours after the onset of stationary phase; lane 3 – SpoIIE-YPet produced after induction with 0.1% xylose, when cells were harvested after 2.5 hours of growth; lane 4 – SpoIIE-YPet produced after induction with 0.5% xylose, when cells were harvested after 2.5 hours of growth. SpoIIE-YPet is highly degraded. This may have occurred during sample preparation or it may be subject to proteolysis in both vegetative and sporulating cells.

Video S1. Localization of RefZ and SpoIIE. Time laps localization of SpoIIE-mScarlet (red) and RefZ-mGFP (green) in a sporulating culture of strain IB1823. The video shows an overlay of the GFP and Scarlet signals. The frames (nine in total) were taken sequentially every 10 minutes. Refer also to details from Fig. 4C.

References

16. Barák, I., Behari, J., Olmedo, G., Guzmán, P., Brown, D. P., Castro, E., Walker, D. E., Westpheling, J., and Youngman, P. (1996) Structure and function of the Bacillus SpolIE protein and its localization to sites of sporulation septum assembly. *Mol. Microbiol.* **19**, 1047–1060

23. Barák, I., and Youngman, P. (1996) SpoIIE mutants of Bacillus subtilis comprise two distinct phenotypic classes consistent with a dual functional role for the SpoIIE protein. *J. Bacteriol.* **178**, 4984–4989

31. Karimova, G., Pidoux, J., Ullmann, A., and Ladant, D. (1998) A bacterial two-hybrid system based on a reconstituted signal transduction pathway. *Proc. Natl. Acad. Sci. U. S. A.* **95**, 5752–5756

34. Muchová, K., Chromiková, Z., Bradshaw, N., Wilkinson, A.J., and Barák, I. (2016) Morphogenic Protein RodZ Interacts with Sporulation Specific SpoIIE in *Bacillus subtilis*. *PLoS One* **11**, e0159076

47. Youngman, P., Perkins, J.B., and Losick, R. (1984) Construction of a cloning site near one end of Tn917 into which foreign DNA may be inserted without affecting transposition in Bacillus subtilis or expression of the transposon-borne erm gene. *Plasmid* **12**, 1–9

48. Backman, K., Ptashne, M., and Gilbert, W. (1976) Construction of plasmids carrying the cI gene of bacteriophage lambda. *Proc. Natl. Acad. Sci. U. S. A.* **73**, 4174–8

49. Lewis, P.J., and Marston, A.L. (1999) GFP vectors for controlled expression and dual labelling of protein fusions in *Bacillus subtilis*. *Gene* **227**, 101–109

51. Jamroškovič, J., Pavlendová, N., Muchová, K., Wilkinson, A.J., and Barák, I. (2012) An oscillating Min system in *Bacillus subtilis* influences asymmetrical septation during sporulation. *Microbiology* **158**, 1972–81

52. Ju, J.;Luo, T. and Haldenwang, W.G. (1998) Forespore expression and processing of the SigE transcription factor in wild-type and mutant *Bacillus subtilis*. *J. Bacteriol.* **180**, 1673–1681

53. Yanisch-Perron, C.; Vieira, J., and Messing, J. (1985) Improved M13 phage cloning vectors and host strains: nucleotide sequences of the M13mp18 and pUC19 vectors.

Gene **33**, 103–119
